# ﻿A new species of *Petalacmis* firefly from Bolivia, with a key to species (Coleoptera, Lampyridae)

**DOI:** 10.3897/zookeys.1092.80464

**Published:** 2022-04-04

**Authors:** Luiz F. Lima da Silveira, Marc A. Branham

**Affiliations:** 1 Department of Biology, Western Carolina University, Cullowhee, NC 28723, USA Western Carolina University Cullowhee United States of America; 2 Department of Entomology and Nematology, University of Florida, P.O. Box 110620, Gainesville, FL 32611-0620, USA University of Florida Gainesville United States of America

**Keywords:** Firefly, Lampyrini

## Abstract

*Petalacmis* Olivier, 1908 is a poorly known genus of firefly endemic to South America and is currently the only member of the subfamily Lampyrinae, tribe Lampyrini known to occur on the continent. Here, we describe a new species, *Petalacmistriplehorni***sp. nov.** from Bolivia and compare it to the two other described species in the genus. A key to *Petalacmis* species based on male traits, as well as illustrations of morphological features, are given in detail for the first time. We present unique, previously neglected traits of *Petalacmis* species and compare them to other Lampyrinae.

## ﻿Introduction

*Petalacmis* E. Olivier, 1908 is an interesting and unique genus of fireflies (Coleoptera, Lampyridae) with distinctive antennal morphology: males have only nine antennomeres, the ninth very elongate and paddle-shaped. *Petalacmis* is poorly represented even in large collections worldwide (LS and MB pers. obs.), and even basic aspects of its morphology are lacking due to the rarity of specimens available for dissection. In fact, this genus is only known from male specimens, a widespread phenomenon in lampyrids (Silveira and Mermudes 2013, 2014; Ferreira et al. 2019, 2020; Bocakova et al. 2022) and in elateroids as a whole (e.g., Bocak et al. 2016; Biffi et al. 2021). Therefore, detailed studies on the diversity of *Petalacmis* species are greatly needed in order to produce a more comprehensive understanding of the family Lampyridae, particularly in the tribe Lampyrini. Astonishingly, *Petalacmis* is the only known genus of its tribe known to occur in South America, where it remains more poorly known than its counterparts in both the Old World and North America.

*Petalacmis* was erected for its type species, *Petalacmispraeclarus* E. Olivier, 1908, with no subfamilial placement, by Olivier (1908). In a later work, Olivier (1910) placed the genus in the subfamily Photininae. In his 1959 work, Green moved *Petalacmis* to the superfamily Lampyrinae and the tribe Lampyrini. Green’s placement of *Petalacmis* was followed in McDermott’s subsequent taxonomic work on Lampyridae (McDermott 1964) and his 1966 catalog. Phylogenetic analyses consistently placed *Petalacmis* in the subfamily Lampyrinae, but its affinities remain unsteady. Phylogenetic analyses based on morphological data recovered *Petalacmis* close to the Neotropical Pleotomini Summers, 1875 (Jeng 2008), whereas molecular-based phylogenies found it closer to part of Lamprocerini Olivier, 1907 (Martin et al. 2019). The most recent comprehensive classification places *Petalacmis* in Lampyrini (Martin et al. 2019).

*Petalacmis* currently consists of two species: *P.praeclarus* from Brazil, Bolivia, and Peru, and *Petalacmiswittmeri* Reichardt, 1963 known only from Brazil. A third species, *Petalacmistriplehorni* sp. nov. only known from Bolivia, is described here. We provide the first identification key to *Petalacmis* species based on male morphology and document the morphological features of this genus for the first time. A discussion of *Petalacmis* morphology and its comparison to other Lampyrinae is presented.

## ﻿Materials and methods

Specimens were both studied and imaged under dissection microscope Leica M205 C. Digital images were obtained and stacked using the Leica Application Suite X. Specimens of *P.praeclarus* and *P.triplehorni* sp. nov. were measured under a Leica MZ16 microscope with a calibrated eyepiece graticule, and measurements were converted to millimeters (Table 1). A whole specimen, as well as the abdomen of a second were soaked in KOH 10% for 24 h before dissection to digest soft tissues. The classification scheme used in this study follows Martin et al. (2019), morphological terminology follows Silveira and Mermudes (2014), and wing venation nomenclature follows Lawrence et al. (2021). Specimens were deposited at the following institutions: Museo Nacional de Historia Natural, La Paz, Bolivia (**ANCB**; J. Tavel); Division of Plant Industry, Florida State Collection of Arthropods, Gainesville, Florida, United States of America (**FSCA**; P. Skelley); United States of America National Museum of Natural History, Washington, DC, USA (**USNM**; M. Branham), University of Georgia Collection of Arthropods, Athens, Georgia, USA (**UGCA**; J. McHugh); Ohio State University, C.A. Triplehorn Insect Collection, Columbus, Ohio, USA (**OSUC**; L. Musetti).

**Table 1.** Comparative measurements (average, range between parentheses) between the three known species of *Petalacmis*. Measurements were taken from the material examined (see above), except for those of *P.wittmeri*, which were taken from Reichardt (1963).

Dimensions (mm)	*P.praeclarus* (*n* = 8)	*P.wittmeri* (*n* = 1)	*P.triplehorni* (*n* = 15)
Total Length	9.01 (8.13–9.46)	6.8	5.34 (5.06–5.81)
Pronotal Length	2.04 (1.74–2.24)	1.5	0.99 (0.91–1.07)
Elytral Length	7.02 (6.39–7.3)	5.3	4.35 (4.15–4.73)

## ﻿Results

### ﻿Taxonomy

**Lampyridae: Lampyrinae: Lampyrini**

#### ﻿ Petalacmis triplehorni

Taxon classificationAnimaliaColeopteraLampyridae

Silveira & Branham sp. nov.

B9FFA2FA-DE3A-5987-8D51-A569F262F2F7

http://zoobank.org/2F8DCDAF-6A31-48BE-B757-74A91AFCDA37

**Figure 1.**
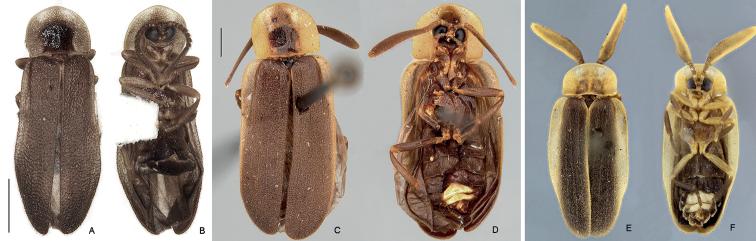
Habiti of *Petalacmis* species **A, B***P.triplehorni* sp. nov., holotype (male, prior to dissection), habitus **A** dorsal **B** ventral **B, C***P.praeclarus* (male, from Piracicaba, São Paulo) **B** dorsal **C** ventral **E, F***P.wittmeri* holotype (male), habitus **E** dorsal **F** ventral. Scale bars: 1 mm (**A–D**).

**Figure 2.**
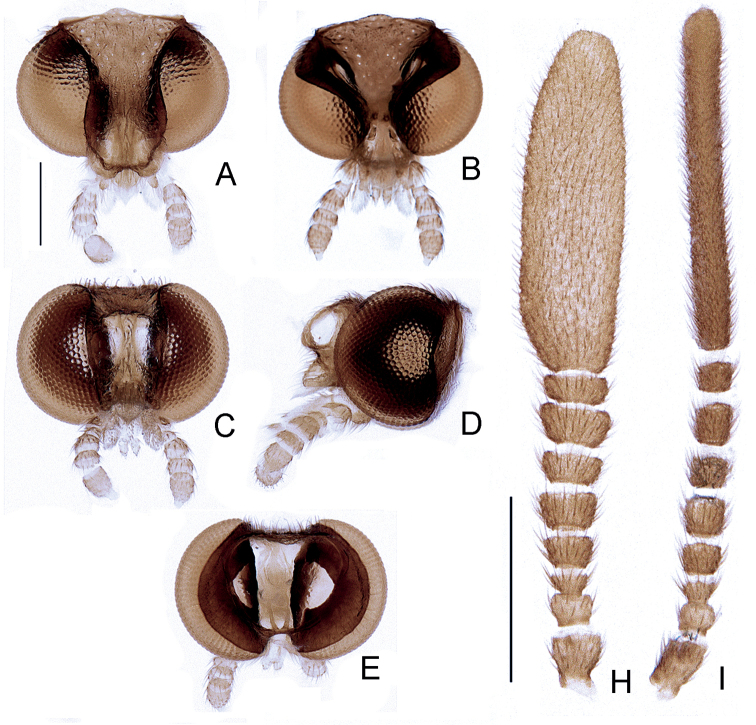
*Petalacmistriplehorni* sp. nov., male head **A–F** core head **A** dorsal **B** ventral/occipital **C** frontal **D** lateral **E** posterior **H, I** antenna **H** lateral **I** frontal. Scale bars: 250 µm (**A–E**); 500 µm (**H, I**).

**Figure 3.**
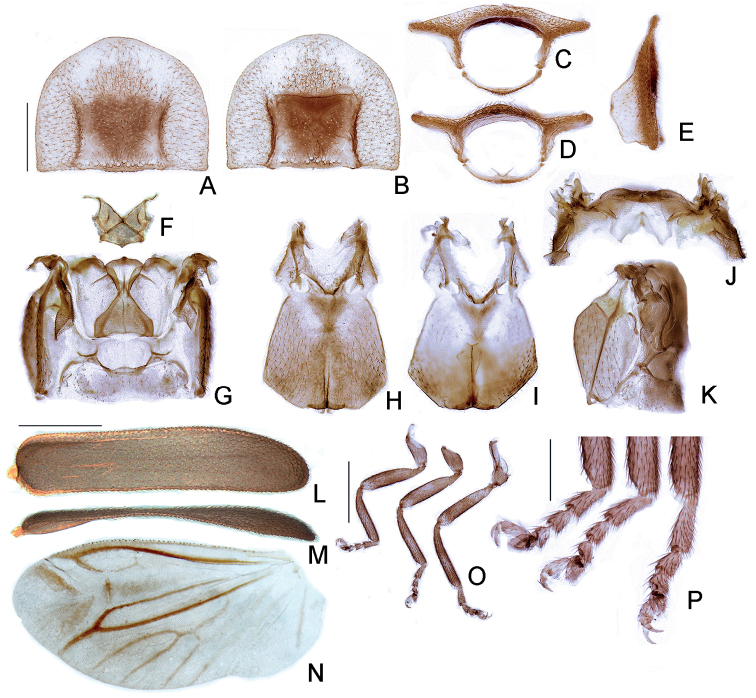
*Petalacmistriplehorni* sp. nov., male thorax. **A–E** prothorax: **A** dorsal **B** ventral **C** anterior **D** posterior **E** lateral. **F–K** pterothorax: **F** mesoscutellum, dorsal **G** alinotum, dorsal **H** pterothorax, ventral **I** pterothorax, dorsal (detail of meso and metaendosterna **J** alinotum, dorsal **K** pterothorax and abdominal tergum I, lateral **L–N** wings: **L** elytron, dorsal **M** elytron, lateral (outer view) **N** left wing, dorsal **O, P** legs: **O** left pro, meso and metaleg (left to right) **P** detail of left pro, meso and metaleg apices (left to right). Scale bars: 500 µm (**A–K**); 1 mm **L–N**; 750 µm (**O**); 250 µm (**P**).

**Figure 4.**
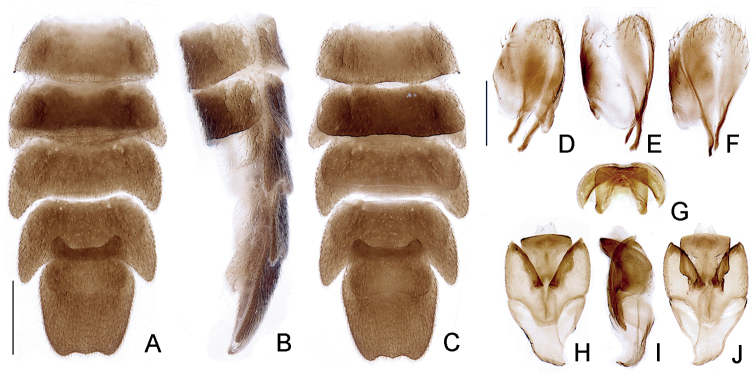
*Petalacmistriplehorni* sp. nov., male abdomen **A–C** core abdomen (segments IV–VIII) **A** dorsal **B** lateral **C** ventral **D–F** aedeagal sheath **D** dorsal **E** lateral **F** ventral **G–J** aedeagus (distal tips oriented toward top of the figure) **G** apical/posterior **H** dorsal **I** lateral, **J** ventral. Scale bars: 500 µm (**A–C**); 250 µm (**D–I**).

Figs 1 , 2 , 3 , 4 ; Suppl. material 1: Fig. S1

##### Diagnosis.

The three species of *Petalacmis* are easily diagnosable by size (Table 1), along with the morphology of antennae, elytra, and pygidium (see key below). *Petalacmistriplehorni* sp. nov. can be identified by the following combination of characters: antennomeres V and VII slightly longer and wider than adjacent antennomeres (Fig. 2H–I), elytron subparallel-sided (Fig. 3L), pygidium with sides divergent to basal third, then convergent apically with almost straight margins, posterior margin slightly bisinuose, lateral thirds subequal to or slightly longer than median third (Fig. 4A, C). Measurements are given in Table 1.

##### Description of male.

Color pattern: overall brown, except for the dark brown pronotal disc, translucent parasagittal rounded spots on pronotal expansions, and translucent sterna VI–VIII (Fig. 1); pronotal expansions often light brown (Suppl. material 1: Fig. S1).

***Head***: head capsule about 1/3× wider than long, posterior margin almost straight, except for the dorsal margin of occiput, which is rounded (see dorsal view, Fig. 2A), slightly taller than long (Fig. 2D), vertex slightly depressed between the eyes (Fig. 2E). Frons slightly intumescent (Fig. 2D), antennal sockets elliptical, 2× taller than wide, obliquely disposed, as wide as 1/3 eye; antennifer process barely visible (Fig. 2C). Eye as wide as 1/3 head width in dorsal view (Fig. 2A), 2/5 in ventral view (Fig. 2B), dorsal margin emarginated inwards (Fig. 2A), frontal inner margin rounded, strongly convergent ventrally (Fig. 2B), almost occupying the whole head capsule in lateral view (Fig. 2D), indented posteriorly (Fig. 2B, D). Antenna with nine antennomeres (Fig. 2H, I); scape slightly longer than wide, basally constricted; pedicel basally constricted, slightly wider than long, 1/2 as long as scape; antennomeres III–VIII transverse, progressively compressed, with decumbent bristles, subequal in length, except for V and VII, which are slightly longer and wider than neighboring antennomeres, IX petal-shaped, lateral margins asymmetrical (Fig. 2H); frontoclypeus strongly depressed between antennal sockets and labrum (Fig. 2C). Labrum (Fig. 2A–C) subcircular, slightly acuminate anteriorly, connate to frontoclypeus, slightly wider than antennal socket. Mandibles short (as long as labrum), slightly curved, apically obtuse, homogeneously bristled (Fig. 2A, D). Maxilla (Fig. 2B, C) with cardo well sclerotized, rectangular; stipes oblong, subtriangular in ventral view, internal margin slightly curved, posterior margins rounded, palp with 4 palpomeres; II–IV subcylindrical and transverse, II longer than I and as long as palpifer, I as long as III, III transverse; IV lanceolate, 3× longer than III, with apical margin covered with bristles. Labium (Fig. 2B) with mentum membranous and barely distinct, divided sagittally forming two plates, each plate elongate; submentum membranous and indistinct; palp with 2 distinct palpomeres, apical palpomere obconical. Gula coriaceous, as long as wide, paired tentorial pits conspicuous. Occiput subtriangular, maximum width slightly over 1/2 head width, anterior margin slightly sinuose (Fig. 2E).

***Thorax***: pronotum with anterior margin slightly sinuose and acuminate anteriorly (Fig. 3A), lateral margins slightly rounded to subparallel, posterior angles somewhat acute but not pointed, posterior margin almost straight, slightly rounded by the disc; disc subquadrate (Fig. 3A), slightly depressed by posterior half (in lateral view, Fig. 3E), regularly punctured, punctures small and bristled, evenly spaced about 2× puncture size; without a distinct line of deeper marginal punctures; pronotal expansions well developed, anterior expansion convex in lateral view (Fig. 3C, E), maximal length almost as long as disc (Fig. 3A); posterior margin about as wide as distance between elytral humeri (Fig. 1A); lateral expansions bent upwards in posterior view (Fig. 3D). Hypomeron slightly over 2× longer than tall, with a well-delimited posterior angle (Fig. 3E). Prosternum smallest length about 10× as wide as its greatest length (Fig. 3B). Proendosternite apically acute, widely divergent, as long as core prosternum smallest length (Fig. 3D). Mesoscutellum very short (Fig. 3F), with posterior margin pointed, normally at a lower level than elytra (Fig. 1A). Elytron (Fig. 3L, M) subparallel-sided, almost 5× longer than wide, pubescent, secondary pubescence absent, with shallow irregular punctures, texturized, with evanescent 4 costae, marginal costa narrow, epipleuron reaching basal ¼. Hind wing well developed (Fig. 3N), posterior margin with anal embayment (sensu Lawrence et al. 2021), slightly less than 2× wider than long, r4 3× longer than r3, radial cell 3× wider than long, distant from anterior margin more than the caliper of RA, costal row of setae conspicuous (Fig. 3N); CuA1 cross vein evanescent, CuA3+4 cross vein absent; radial cell, r3 and r4 evanescent, veins posterior to MP progressively evanescent from apex to base.

Alinotum overall weakly sclerotized, slightly wider than long (Fig. 3G), lateral margins convergent posteriorly, posterior margin slightly emarginate; prescutum extending up to half metascutum length; without a distinct rounded area, scutum-prescutal plates distinct and extending ridges to half alinotum length; metascutellum glabrous, with lateral margins subparallel-sided, scutum-scutellar ridge strongly divergent posteriorly. Mesosternum weakly sclerotized, posterior margin medially rounded (Fig. 3H). Mesosternum-mesepisternum suture barely visible (Fig. 3H). Mesepisternum-mesepimeron suture conspicuous (Fig. 3H). Mesepimeron-metasternum suture coriaceous (Fig. 3H, I). Metasternum strongly depressed by mesocoxae, without a distinct anterior medial keel, discrimen reaching basal 1/3 of metasternum length, lateral margins divergent posteriorly up to outermost part of metacoxa, then convergent posteriorly, posterior margin bisinuose (Fig. 3H–I). Metepisternum almost 3× longer than tall Fig. (3K). Profemur about as long as protibia; meso and metatibia of about the same length and slightly longer than protibia (Fig. 3O). All legs lacking tibial spurs, with tarsomeres progressively shorter up to IV, which is bilobated, lobes reaching ½ V length, V slightly shorter than I, and with simple, untoothed claws (Fig. 3O, P). Mesendosternum with 2 parasagittal projections slightly directed outwards, irregularly alate (Fig. 3I–K). Metendosternum spatulate, roughly rhomboid (about as long as wide, with 2 acute lateral laminae), anteriorly indented (Fig. 3I).

***Abdomen***: tergum I with laterotergite membranous, roughly triangular, almost indistinct (Fig. 3K); spiracle elliptical, obliquely attached to thorax (Fig. 3G, K). Posterior corners of terga I–III almost right-angled, IV slightly projected, V–VII rounded and progressively projected and acute (Fig. 4A–C). Sterna II–IX visible (Figs 1B, 4C), V distinctly more sclerotized than neighboring sterna, VI and VII with well-developed, transverse light organs, almost as long and wide as sterna (Fig. 4B, C, Suppl. material 1: Fig. S1). Spiracles ventral, at mid-length (Fig. 4B, C). Sternum VIII with posterior margin slightly emarginate (Fig. 4B, C). Pygidium with anterior margin strongly emarginate, lateral margins almost straight and convergent posteriorly, posterior margin bisinuate, lateral thirds subequal to or slightly longer than median third (Fig. 4C). Syntergite membranous, as long as 4/5 sternum IX length, widely connate to sternum IX, without distinct sutures, posteriorly bristled, anterior margin slightly emarginate (Fig. 4D–F). Sternum IX symmetric, medially divided by a membranous line, anterior margin rounded, lateral margins strongly convergent anteriorly (Fig. 4D–F). Aedeagus overall well sclerotized (Fig. 4G–J). Phallus (Fig. 4G–I) with a well-sclerotized dorsal plate, ventral plate indistinct; dorsal plate basally connate to parameres, curved dorsally, apically truncate, deeply excavate ventrally (Fig. 4G), without apical lobes or arrow-shaped structures. Parameres ventrally projected basally, projection somewhat rounded; reaching the dorsal plate of phallus length, with typical lampyrine inner lobes but coriaceous; with an apical pointed projection, which is membranous.

**Figure 5.**
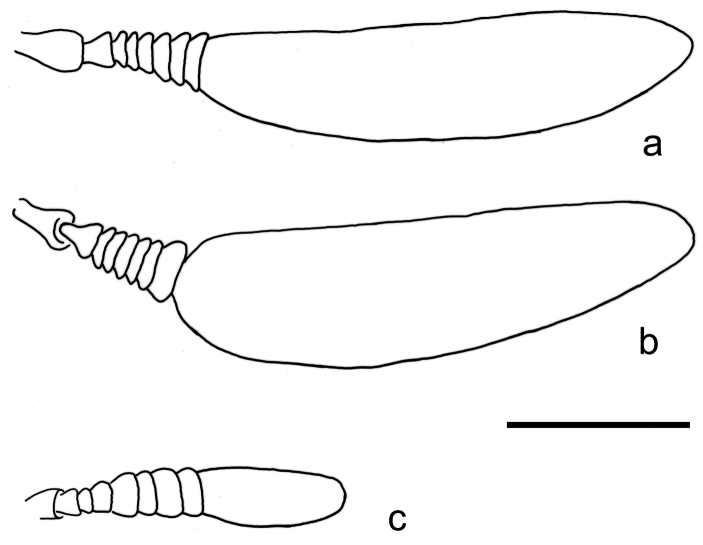
Comparison between the antennal morphologies **A***Petalacmispraeclarus***B***P.wittmeri***C***P.triplehorni* sp. nov. Scale bar: 1.0 mm.

##### Female and immature stages.

Unknown.

##### Etymology.

This species is named for Dr Charles “Chuck” Triplehorn, Professor Emeritus of the Ohio State University, who collected the first specimens of *Petalacmispraeclarus* that one of us (MAB) first encountered as a graduate student while investigating the systematics of the family Lampyridae. Dr Triplehorn has been both a mentor and an inspiration to MAB. It is with great appreciation and respect for Dr Triplehorn that we name this species after him.

##### Material examined.

***Holotype***, male. Bolivia: Santa Cruz, Potrerillo del Guendá Reserve, 1322’ elev., 17°40.262'S, 63°27.445'W, at light, J. McHugh lab exped. leg., 6–12-I-2005 (ANCB).

***Paratypes*** (*n* = 14). Bolivia • Santa Cruz, Potrerillo del Guendá Reserve.; 1322’ elev.; 17°40.262'S, 63°27.445'W; at light; J. McHugh lab exped. leg.; 6–12-I-2005 (1 ♂, USNM) • Santa Cruz, 3.7 km SSE of Buena Vista, Hotel Flora and Fauna; 405 m elev.; 5–15-XI-2001; 17°29.949'S, 63°33.152'W; M.C. Thomas & B.K. Dozier leg.; tropical transition forest (3 ♂, FSCA) • idem. (2 ♂, ANCB); Santa Cruz, 3.7 km SSE of Buena Vista, Hotel Flora and Fauna; 405 m elev.; 5–15-XI-2001, 17°29.949'S, 63°33.152'W; M.C. Thomas & B.K. Dozier leg.; tropical transition forest (1 ♂, OSUC) • Santa Cruz, 3.7 km SSE of Buena Vista, Hotel Flora and Fauna; 405 m elev.; 5–15-XI-2001; 17°29.949'S, 63°33.152'W; M.C. Thomas & B.K. Dozier leg.; tropical transition forest, blacklight trap (2 ♂, UGCA) • Santa Cruz, 3.7 km SSE of Buena Vista, Hotel Flora and Fauna; 430 m elev.; 2–13-III-2000; M.C. Thomas leg.; tropical transition forest (3 ♂, ANCB) • Santa Cruz, 3.7 km SSE of Buena Vista, Hotel Flora and Fauna; 430 m elev.; 14–19-X-2000; M.C. Thomas leg.; tropical transition forest (1 ♂, ANCB) • Santa Cruz, 40 km NW of Potrerillo del Guendá; 400 m elev.; Gino Nearns leg., 17-XII-2004 (1 male, USNM).

### ﻿Key to the species of *Petalacmis*

**Table:** 

1	Elytron subparallel-sided, slightly tapering distally (Fig. 1A–D)	**2**
–	Elytra elliptical, widest in middle (Fig. 1E, F)	***Petalacmiswittmeri* Reichardt**
2	(1). Antennomeres V and VII as wide as VI and VIII, apical antennomere nearly 2× longer than remaining antennomeres together (Fig. 5A); elytral outer expansion (also known as explanate margin) extending up to 2/3 of elytral length, yellowish (Fig. 3C, D); pygidium with sides rounded, posterior margin strongly bisinuose, slightly longer at median 1/3	***Petalacmispraeclarus* E. Olivier**
–	Antennomeres V and VII slightly longer and wider than neighbor antennomeres, apical antennomere as long as the remaining antennomeres together (Fig. 2H, I); elytral outer expansion feebly developed throughout, and of the same color as the rest of the elytron (brown) or slightly brighter (Figs 1A, B, 3L; Suppl. material 1: Fig. S1A); pygidium with sides almost straight, posterior margin slightly bisinuose, lateral thirds subequal to or slightly longer than median 1/3 (Fig. 4A–C)	***Petalacmistriplehorni* sp. nov.**

#### ﻿ Petalacmis praeclarus

Taxon classificationAnimaliaColeopteraLampyridae

Olivier, 1908

83463A79-C782-59BB-9988-98C6AECA671A

##### Material examined.

Brazil: Minas Gerais, Lambari [spelled Lambary], XI.1924, J. Halik col. (1 male, USNM 3083) // Brazil Halik 1966 coll.; same data, XI.1924 (1 male, USNM 3084); same data, XI.1924 (1 male, USNM 3085); São Paulo, São Paulo, Santana [spelled St. Anna, Cap. S. Paulo], XII.1934, J. Halik col. (1 male, USNM 5047).] // Brazil Halik 1966 coll. collection; São Paulo, Cantareira, 23.VII.1933, J. Halik col. (1 male, USNM 2208) // Brazil Halik 1966 coll. collection; São Paulo, Botanical garden [spelled *horto flor.*], XII.1921, J. Halik col. (1 male, USNM 7190) // Brazil Halik 1966 coll. collection; São Paulo, Piracicaba, 6.X.1965, Blacklight, C. A. Triplehorn col. (2 males, M. Branham collection).

## ﻿Discussion

### ﻿Distribution of *Petalacmis* spp.

To date, all *Petalacmis* species are known from lowland localities across South America east of the Andes. *Petalacmispraeclarus* was described from “Brazil”. It has since been collected in Bolivia and Peru (Olivier 1908; Reichardt 1963; McDermott 1964, 1966; Lawrence et al. 2001) and the Atlantic Rainforest in Brazil (see Material examined above). Such a widespread distribution is uncommon in Neotropical fireflies, and the existence of overlooked or cryptic species should be considered in future comprehensive taxonomic reviews. In 1963, Reichardt described the second species, *P.wittmeri* which was collected Ananindeua, in Pará state, Brazil. *Petalacmistriplehorni* sp. nov. was collected near Buena Vista, Santa Cruz, Bolivia. Due to its small size and nocturnal habit, the genus is likely to have been overlooked, particularly in South America, where taxonomic expertise in fireflies was largely lacking until fairly recently.

### ﻿Thoughts on the mating system of *Petalacmis* species

Due to the presence of large eyes and photic organs in the male, one might expect that both the male and female of these species are luminous and use luminous signals for pair-formation, as seen in several firefly subfamilies (Branham and Wenzel 2003; Branham 2010; Stanger-Hall et al. 2018). The fact that no females are currently known for any of the three species in this genus may suggest that females are sedentary, perhaps brachypterous or even apterous, as seen in other lampyrids (e.g., Faust 2017; Stanger-Hall et al. 2018). Alternatively, these species may be so uncommonly encountered that no females have been collected.

### ﻿Morphology and systematics of *Petalacmis*

The affinities of *Petalacmis* have been investigated in two comprehensive phylogenies of Lampyridae. Based on morphological data, Jeng (2008) found Neotropical Pleotomini Summers, 1875 (i.e., *Calyptocephalus* Gray, 1832, *Phaenolis* Gorham, 1880, *Ophoelis* Olivier, 1911, and *Roleta* McDermott, 1962) sister to *Petalacmis*. On the other hand, the molecular-based phylogeny by Martin et al (2019) found *Petalacmis* sister to *Lucio* Laporte, 1833 and *Lamprocera* Laporte, 1833, both in the Lamprocerini Olivier, 1907. Neither phylogenetic hypothesis found evidence of exclusive shared ancestry with the Lampyrini, where they are currently placed (Martin et al. 2019). However, the taxa which were more closely associated with *Petalacmis* by Jeng (2008) were not included in Martin et al. (2019); hence, the sister-lineage of *Petalacmis* remains unclear.

As our study was the first to thoroughly survey the anatomy of *Petalacmis*, we provide some comparisons to inform the ongoing debate on the phylogenetic affinities of this genus. We assume that our description of *P.triplehorni* sp. nov. includes traits that are likely to be shared with other species in the genus, or even traits common to all of them. In addition to the very distinctive “petal-like” antennal morphology, we observed previously obscured traits of *Petalacmis* that differ significantly from those of other lampyrine genera. For example, *Petalacmis* is unique among lampyrine genera in having (i) an intumescent and laterally keeled frons (Fig. 2A, D), (ii) mandibles short apically rounded (i.e., obtuse, not pointed) frons (Fig. 2A, C–D), and (iii) a dorsal plate of the phallus much shorter than the phallobase and strongly bent dorsally (in ventral view), with sides parallel and straight in apical view, deeply grooved (in apical/posterior view), and apically truncate (Fig. 4H–J). Other genera of Lampyrini often have frons that are not intumescent (e.g., flat or depressed between antennal sockets), mandibles apically very acute (i.e., needle-like), and phalli at least slightly longer than phallobase, sinuose or almost straight (but never strongly bent dorsally), with sides sinuose, and apex variably acute, often with arrow-shaped apices (e.g., Green 1959; Geisthardt 1986; Kazantsev 2010; Constantin 2014). Interestingly, the intumescent frons seen in *Petalacmis* is known in at least some Pleotomini (e.g., *Roleta*; Jeng et al. 2006), and in the amydetine taxa *Magnoculus* and *Memoan* (Silveira and Mermudes 2013; Campello-Gonçalves et al. in press).

Another trait shared with all three amydetine genera (*Amydetes* Illiger, 1807, *Magnoculus* McDermott, 1964, *Memoan* Silveira & Mermudes, 2013; Campello-Gonçalves et al. in press), as well as many other glowing firefly taxa (*Phausis* LeConte, 1851, *Lamprohiza* Motschulsky, 1853) is the more sclerotized and slightly emarginated sternum V (Fig. 4C), which precedes the lanterns. Another feature of *P.triplehorni* sp. nov. that catches the eye is the shape of abdominal sternum IX (Fig. 4F), which is medially divided by a membranous line—a typical trait of the Lampyrini (e.g., Kazantsev 2010) that is also commonly observed across Lamprocerini and Cratomorphini (e.g., Campos et al. 2018; Silveira et al. 2019; Lima et al. 2021), but seldom seen in Photinini (e.g., Silveira et al. 2022).

Concerning aedeagal morphology, lampyrine taxa very often have parameres with an elongate, membranous apex—a trait also commonly found across Lamprocerini and Cratomorphini (e.g., Campos et al. 2018; Silveira et al. 2019). The same membranous apex is found on the aedeagus of *P.triplehorni* sp. nov., but in a rudimentary form (Fig. 4). The typical inner lobe of parameres (as seen for example in *Lampyris* (e.g., Geisthardt 1986; Kazantsev 2010; Constantin 2014) is also present in *P.triplehorni* sp. nov., although distinctly thinner and less sclerotized.

Recently, it has become more common that taxonomic studies report detailed exo- and endoskeletal traits of the thorax, like the shape of meso and metatergal ridges, as well as those of the endosternites. Currently, no such information is available for any taxa of Lampyrini, which hampers any comparison. Nevertheless, we observed some interesting traits in *Petalacmistriplehorni* sp. nov. and compare it to known lampyrine taxa. For instance, the mesoscutellum of *P.triplehorni* sp. nov. is so reduced that it is almost triangular, despite the median pointed projection at the posterior margin (Fig. 3F). To our knowledge, no such rudimentary shape has been observed before in any other lampyrid. Moreover, no other Lampyrini is known to possess an extremely reduced and posteriorly pointed mesoscutellum. However, we observed a rather pointed mesoscutellum in the lamprocerine genus *Tenaspis* (e.g., *T.angularis*, *T.sinuosa*; LFLS pers. obs.).

The alinotum of *P.triplehorni* sp. nov. is clearly distinct, with a scutum–prescutal ridge that extends to less than half the length of the metanotum (Fig. 3G), otherwise reaching or almost so the posterior margin in most other lampyrids, where known (e.g., Silveira et al. 2019). Such a short scutum–prescutal ridge is only known in the distantly related Amydetinae (Silveira and Mermudes 2014; Campello-Gonçalves et al. in press). The function of that modified alinotum in Lampyridae remains unknown, but it likely reflects changes in the flight muscles that attach to these ridges, and possibly associated with changes in flight pattern. The metascutelum of *P.triplehorni* sp. nov. has rather oblique anterior ridges (about 45° to the lateral ridges), otherwise almost transverse as found in most other lampyrids (e.g., Silveira et al. 2019). It is also noteworthy that *P.triplehorni* sp. nov. has a metaendosternum with anterior margin wide and emarginate (a trait so far only observed in Cratomorphini; Campos et al. 2018).

*Petalacmis* shares with Lampyrini, Pletomonini, and Lamprocerini, the ventral position of abdominal spiracles, as well as the reduced mandibles. However, this genus lacks key traits of all these three tribes. For example: both *Lucio* and *Lamprocera*, the taxa placed close to *Petalacmis* in Martin et al.’s (2019) phylogenetic analysis, have biflabellated antennae, elytra broadly expanded laterally and bent ventrally, and a transverse pygidium. In contrast, *Petalacmis* has a petal-shaped antenna, elytra feebly expanded and almost flat in lateral view (not bent ventrally), and a pygidium much longer than wide. *Petalacmis* also lacks the typical biflabellate antennae, and the bilobate sternum VIII, of the Neotropical Pleotomini. Yet, *Petalacmis* and the Neotropical Pleotomini share an intumescent frons, and an elongate pygidium. Lampyrini taxa usually have a transverse pygidium (elongate in *Petalacmis*), and their parameres have a well-developed apical membranous projection (e.g., Geisthardt 1986; Kazantsev 2010; Constantin 2014), which is at best rudimentary in *Petalacmis*. Therefore, the unique combination of characters seen in *Petalacmis* makes its taxonomic placement within the family Lampyridae challenging. A well-substantiated tribal placement of *Petalacmis* in Lampyrinae would benefit from its inclusion in future phylogenetic analyses with expanded taxon sampling across the Lampyridae.

Taken together, these observations highlight the potential value of traits typically neglected in lampyrid taxonomy, and invite future anatomical studies concerning the Lampyrinae, particularly the Lampyrini. We hope that our study fosters a future comprehensive review of this interesting firefly genus.

## Supplementary Material

XML Treatment for Petalacmis triplehorni

XML Treatment for Petalacmis praeclarus

## Supplementary materials

Supplementary material 1 Figure S1 Data type Figure Explanation note *Petalacmistriplehorni* sp. nov. habitus of one of the paratypes (note lantern morphology and the brighter pronotal expansions) A dorsal B ventral. https://binary.pensoft.net/file/668156This dataset is made available under the Open Database License (http://opendatacommons.org/licenses/odbl/1.0/). The Open Database License (ODbL) is a license agreement intended to allow users to freely share, modify, and use this Dataset while maintaining this same freedom for others, provided that the original source and author(s) are credited.Luiz F. L. da Silveira, Marc A. Branham
