# Supplementary figures and images for: ﻿A new species of Petalacmis firefly from Bolivia, with a key to species (Coleoptera, Lampyridae)
Source: Zookeys. 2022 Apr 4;1092:63–77. doi: 10.3897/zookeys.1092.80464 (PMC9005466; doi:10.3897/zookeys.1092.80464)

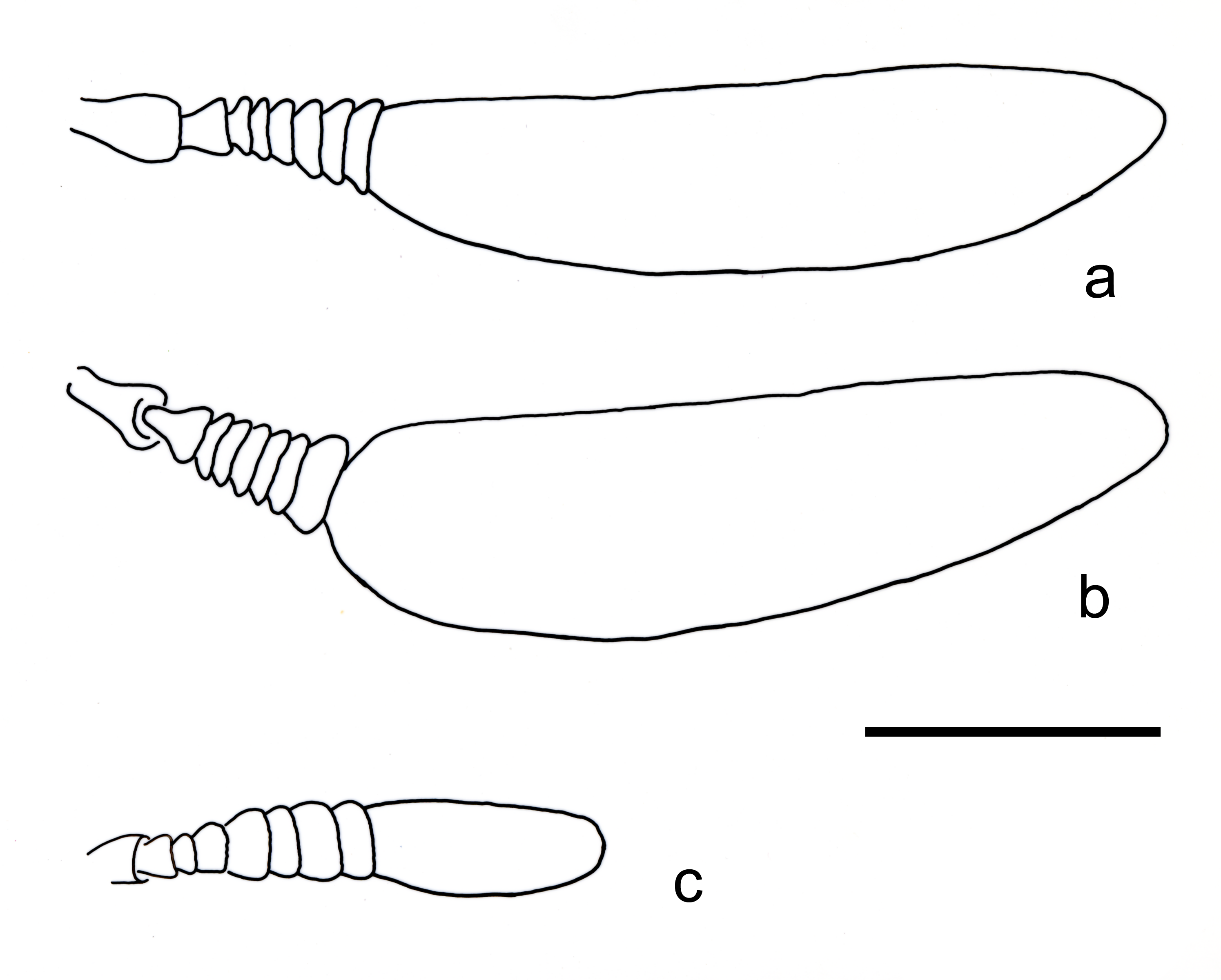

Supplement: Supplementary material 1 — Figure S1 [file zookeys-1092-063-s001.tif]
